# Practical Journalism

**Published:** 1878-11-20

**Authors:** 


### PRACTICAL JOURNALISM.

It has been, and will hereafter be, our continued object to improve and develop the practical feature of our journal. We desire to make it a compendium of all that is practical and useful to the busy practitioner, and we have abundant testimony that our efforts are appreciated in the steady growth o our list, and in the numerous commendatory expressions which come up from the ranks of the profession. We intend that no retrogande step shall be taken in the future conduct of the journal, but rather that it shall improve; and shall more and more conform to the real interests and necessities of progressive and practical medical men. To this end we will endeavor to compass in our gleanings from exchanges the substance of all that is usefbl in both, the home and foreign Journals, and to elicit, from the large number of practical common sense men who make up our list of subscribers, South, West, East and North, such facts, discoveries, practical hints and useful formulæ as are in their possession, and which, from the want of proper encouragement, and from the absence of a medium suited to their taste, have not heretofore been published. That we may be able to carry out this, we invoke the aid of onr medical brethren, particularly of the common sense, hard working, village and country physicians throughout the land. We ask them to send up any and every thing which can, in any degree, interest the profession or benefit medical science.

